# Differential cytopathogenesis of respiratory syncytial virus prototypic and clinical isolates in primary pediatric bronchial epithelial cells

**DOI:** 10.1186/1743-422X-8-43

**Published:** 2011-01-27

**Authors:** Rémi Villenave, Dara O'Donoghue, Surendran Thavagnanam, Olivier Touzelet, Grzegorz Skibinski, Liam G Heaney, James P McKaigue, Peter V Coyle, Michael D Shields, Ultan F Power

**Affiliations:** 1 Centre for Infection & Immunity, School of Medicine, Dentistry & Biomedical Sciences, Queens University Belfast, Belfast BT9 7BL, Northern Ireland; 2 The Royal Belfast Hospital for Sick Children, Belfast BT12 6BA, Northern Ireland; 3 The Regional Virus Laboratory, Belfast Trust, Belfast BT12 6BA, Northern Ireland

## Abstract

**Background:**

Human respiratory syncytial virus (RSV) causes severe respiratory disease in infants. Airway epithelial cells are the principle targets of RSV infection. However, the mechanisms by which it causes disease are poorly understood. Most RSV pathogenesis data are derived using laboratory-adapted prototypic strains. We hypothesized that such strains may be poorly representative of recent clinical isolates in terms of virus/host interactions in primary human bronchial epithelial cells (PBECs).

**Methods:**

To address this hypothesis, we isolated three RSV strains from infants hospitalized with bronchiolitis and compared them with the prototypic RSV A2 in terms of cytopathology, virus growth kinetics and chemokine secretion in infected PBEC monolayers.

**Results:**

RSV A2 rapidly obliterated the PBECs, whereas the clinical isolates caused much less cytopathology. Concomitantly, RSV A2 also grew faster and to higher titers in PBECs. Furthermore, dramatically increased secretion of IP-10 and RANTES was evident following A2 infection compared with the clinical isolates.

**Conclusions:**

The prototypic RSV strain A2 is poorly representative of recent clinical isolates in terms of cytopathogenicity, viral growth kinetics and pro-inflammatory responses induced following infection of PBEC monolayers. Thus, the choice of RSV strain may have important implications for future RSV pathogenesis studies.

## Introduction

Respiratory syncytial virus (RSV) infection is one of the leading causes of infant hospitalization. Virtually all children are infected by the age of two [1]. Due to an incomplete immunization following primary infection [2], re-infections occur throughout life. RSV is also increasingly recognized as a cause of severe illness in adults and especially the elderly [3]. Moreover, the impact of RSV infections is probably underestimated, as early-life infections are associated with the development of recurrent wheeze (asthma) and allergy during childhood [4,5]. Although RSV was first described in 1956 [6], there is still no effective vaccine or specific therapies and treatment is essentially supportive.

Based primarily on G gene variability, RSV strains are divided into subgroups A or B [7]. Many RSV infection experiments employ the A2 strain as the prototype [8]. However, since RSV A2 has been extensively passaged *in vitro *it is likely to have adapted to continuous cell lines and, therefore, might not be representative of recent clinical RSV isolates either genotypically or phenotypically. Moreover, RSV pathogenicity is often investigated in animal models, such as mice, ferrets or cotton rats, which are semi-permissive for RSV infection, and in continuous cells lines *in vitro*, which may not be representative of primary bronchial epithelial cells *in-vivo*. As airway epithelial cells are the principle targets of RSV infection and infants/young children are the most recognizable population affected by severe RSV disease, we hypothesized that an RSV infection model based on primary paediatric bronchial epithelial cells would provide a relevant alternative to more established *in vitro *models.

In the present study, therefore, we investigated RSV infection using primary paediatric bronchial epithelial cells (PBECs), derived from non-bronchoscopic brushings of children undergoing elective surgery [9]. To address the question of whether the prototypic RSV A2 is representative of recent clinical isolates, we isolated 3 viruses, designated RSV BT2a, BT3a and BT4a, from infants hospitalized with bronchiolitis, compared all viruses genetically by sequencing their G genes, and phenotypically by determining the consequences of PBEC infection with each strain on both the cells and the viruses. For most experiments, the clinical isolates were passaged 3 times in HEp-2 cells to limit genetic adaptation to *in vitro *conditions.

Surprisingly, we found that the prototypic A2 strain infected PBECs more efficiently than the 3 clinical isolates and induced dramatic cytopathic effects (CPE), whereas the clinical isolates caused limited CPE. Substantial differences in PBEC infectivity, virus growth kinetics and chemokine secretions, such as interferon-inducible protein 10 (IP-10/CXCL10), regulated upon activation, normal T cell expressed and secreted (RANTES/CCL5), interleukin 6 (IL-6) and IL-8 (CXCL8), were also observed.

These findings indicate that the use of RSV A2 in host-pathogen interaction studies might not be representative of recent RSV clinical isolates in terms of virus growth kinetics, CPE and chemokine induction. They suggest that the choice of RSV strain for further studies should be carefully considered, as recent RSV clinical isolates might reflect more accurately RSV pathogenesis in humans.

## Materials and methods

### Cell line and viruses

HEp-2 cells (kindly supplied by Ralph Tripp, University of Georgia) were cultured in DMEM Glutamax (GIBCO, UK) and 10% FCS supplemented with 50 μg/mL Gentamicin. RSV A2 was kindly supplied by Geraldine Taylor (Institute for Animal Health, UK). The clinical isolates, designated RSV BT2a, BT3a, BT4a, were isolated from infants hospitalized with bronchiolitis in the Royal Belfast Hospital for Sick Children, following parental consent. Briefly, nasal aspirates were added to virus transport medium (DMEM, 25 m*M *HEPES, 50 μg/ml gentamicin, 0.22 *M *sucrose, 30 m*M *MgCl_2_, 0.5 mg/ml fungizone), thoroughly vortexed, sonicated for 10 mins in an ultrasonic water bath (Crest) and centrifugated at 440 × g for 10 min at 4°C. Supernatants were aliquoted and either snap frozen in liquid nitrogen or used directly to inoculate HEp-2 cells, as described below. The identity of each isolate was confirmed by a multiplex virus reverse transcriptase (RT)-PCR strip, as previously described [10].

### Virus culture and growth curves

RSV BT2a, BT3a, BT4a and RSV A2 were cultured in HEp-2 cells as previously described [11], except that DMEM was the medium of choice. To minimize adaptation to HEp-2 cells, RSV BT2a, BT3a and BT4a stocks used for most experiments were derived from three passages in HEp-2 cells, including the initial isolation, before being used to infect the PBECs. Alternatively, to study the consequences of adaptation to HEp-2 cells, a stock of RSV BT2a was generated by passaging 14 times in HEp-2 cells. RSV titers in virus stocks and biological samples were determined as previously described [11], except that DMEM was the medium of choice for virus dilution and infection. Virus titers were calculated by the method of Kärber [12] and reported as log_10 _tissue culture infectious dose 50 (TCID_50_)/mL.

### RSV G gene sequencing

Total viral RNA was extracted from passage 3 virus stocks using TRIzol reagent (Invitrogen) and the G gene of each isolate was amplified in a one-step RT-PCR reaction using RSV G-specific primers (Fw: ACCTCAACATCTCACCATGC; Rev: AGAGTGAGACTGCAGCAAGG) (One step RT-PCR Kit, Quigen). Amplicons were cloned into pXL-TOPO plasmids using the pXL-TOPO cloning kit (Invitrogen) and sequenced using M13 forward and reverse primers, using a Big Dye Terminator v3.1 cycle sequencing kit (Applied Biosystems). Use of all kits followed the manufacturer's instructions. Sequence analyses were performed using Clone Manager suite (Sci Ed Central) and DNA Star Lasergene 8 software (DNASTAR Inc).

### Primary pediatric bronchial epithelial cells (PBECs)

PBECS were obtained from children (1-11 years) undergoing elective surgery at the Royal Belfast Hospital for Sick Children. Children were well and displayed no signs of respiratory viral infection and were clinically free of viral infections for at least one month prior to surgery. Non-bronchoscopic bronchial brushings were performed as previously described [9]. Before inclusion, all samples were confirmed negative for a panel of 12 respiratory viruses using a multiplex virus reverse transcriptase (RT)-PCR strip, as previously described [10]. The cells were seeded in collagen-coated (Purecol^®^, Inamed) 10 cm^2 ^flasks (NUNC) using Airway Epithelial Cell Basal Medium (C-21260) supplemented with Supplement Pack/Airway Epithelial Growth Medium (C-39160) (Promocell medium) (Promocell) at 37°C in 5% CO_2_. Once confluent the cells were passaged into a collagen-coated (Bovine collagen Purecol, INAMED Biomaterials) 75 cm^2 ^flask and expanded at 37°C in 5% CO_2 _until confluent.

### PBEC infection

Expanded PBECs were seeded onto 24 well collagen coated plates (5 × 10^4 ^cells/well) in Promocell medium 24 h before infection. RSV A2, BT2a, BT3a and BT4a stocks were diluted in serum-free DMEM to generate multiplicities of infection (MOI) of 0.1 and 5 in 200 μL, which was applied per well for 2 h at 37°C, 5% CO_2_. For uninfected controls, 200 μL of serum-free DMEM was added/well. Cells infected at MOI 0.1 or 5 were cultured for 96 and 72 h post-infection (hpi), respectively, in 500 μL of culture medium. Infected wells were monitored microscopically for cytopathic effects (CPE) (NIKON ECLIPSE TE-2000 U), while others were fixed for immunofluorescence. Medium (250 μL) was collected at 2 hpi and every 24 hpi for cytokine/chemokine measurements. Cells were scraped into the remaining 250 μL medium, harvested, sonicated, centrifuged at 250 × *g *for 15 min, snap frozen and stored in liquid nitrogen for subsequent virus titration.

### Immunofluoresence

PBECs were rinsed 3 x with 500 μL PBS. Paraformaldehyde (4% v/v) was added/well (500 μL) for 20 min and the PBS washings were repeated. Cells were permeabilised with PBS/0.2% Triton X-100 (v/v) (SIGMA) for 2 h at RT and blocked with 10% non-immune normal goat serum (Zymed, USA) for 30 min. The cultures were stained for RSV F protein (mouse MAb clone 133-1H conjugated with ALEXA 488, Chemicon). Briefly, 200 μL antibody were added to the PBECs for 1 h at 37°C and the cultures were washed 3 x with 500 μL PBS for 15 min at RT. Nuclei were stained using DAPI-mounting medium (Vectashield, Vector Laboratories). Fluorescence was detected by confocal laser scanning microscopy (TCS SP5 Leica).

### Cytokine/Chemokine titrations

Supernatant samples were thawed and analyzed for cytokine/chemokine concentrations using a custom Bio-Plex assay (Bio-Rad, USA) targeting RANTES, IP-10, IL-6, IL-8 (CXCL8), TNF-related apoptosis-inducing ligand (TRAIL) and vascular endothelial growth factor (VEGF), using a Bio-Plex 200 system (Bio-Rad).

### Statistical analysis

All chemokine titration data were analyzed with repeated measures ANOVA with Bonferroni post-test correction for multiple comparisons using Graphpad Prism^® ^5.0. Statistical analyses of growth curves were analyzed with area-under-the-curve measurements followed by a student's paired t-test. Values are presented as the mean ± SE. A *p-value *< 0.05 was considered significant.

### Ethics

This study was approved by The Office for Research Ethics Committees Northern Ireland (ORECNI). All parents gave written consent after receiving appropriate information.

## Results

### Subgroup and genotype analyses of the RSV clinical isolates

**Figure 1:**
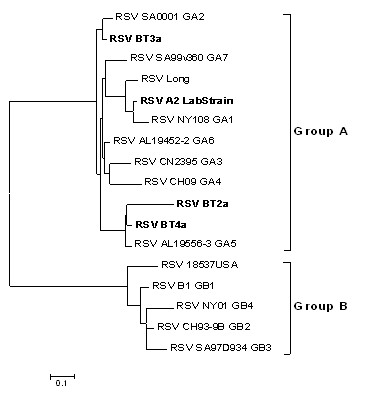
**RSV phylogenic tree**. Alignment of G protein sequences (from aa 212 to the end) was undertaken with Clustal 2.0 software. Sequences were aligned with 9 reference sequences of the subgroup A, including 7 genotypes and the A2 and Long strains. Subgroup B was represented by sequences representative of 5 genotypes. The phylogenic tree was designed using the maximum parsimony algorithm and was drawn with Mega 4.0 software using PHYLIP method. The tree was rooted between subgroups A and B.

The standardized multiplex viral RT-PCR strip [10] indicated that all clinical isolates belonged to RSV subgroup A. This was confirmed by G gene sequencing. Alignments of BT2a, BT3a and BT4a G protein sequences from amino acid 212 to the end of the sequence, along with representatives from each subgroup A and B genotypes, using Clustal 2.0 software determined that the clinical isolates belonged to genotypes GA5 (BT2a and BT4a) and GA2 (BT3a) (Figure 1). In contrast, the prototypic A2 strain belongs to genotype GA1.

### RSV A2 induced more cytopathic effects (CPE) in PBECs than the 3 clinical isolates

**Figure 2:**
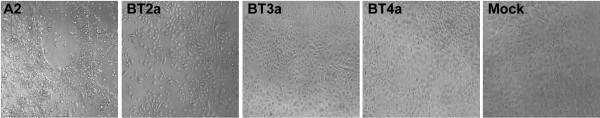
**RSV A2 induces more cytopathic effects (CPE) than the 3 clinical isolates**. PBECs from 5 individual donors were infected with RSV A2, BT2a, BT3a and BT4a (MOI of 0.1) during 2 h and washed with PBS. Seventy two hpi, cells were examined by phase contrast microscopy. Micrographs of a representative field for each RSV strain are presented. PBECs infected with RSV A2 demonstrated substantial CPE, whereas cells infected with RSV BT2a and BT3a displayed only limited CPE. Cells infected with RSV BT4a did not demonstrate any obvious CPE and were similar to non-infected cultures. Original magnification, x10.

To determine the cytopathic consequence of infection on PBECs, duplicate wells from the same donor were infected with RSV A2, BT2a, BT3a, BT4a (n = 5 donors) (MOI = 0.1 or 5) or mock-infected (n = 2 donors) and monitored every 24 h thereafter by phase contrast microscopy. At MOI = 0.1, RSV A2 infection was characterized by large syncytia formation, cell rounding and extensive monolayer disruption over time (Figure 2). In contrast, RSV BT2a and, to a lesser extent, BT3a infection resulted in some cell rounding and limited monolayer disruption. However, there was no evidence of syncytium formation in these cells. Interestingly, RSV BT4a-infected PBECs and mock-infected controls were indistinguishable in terms of CPE. Similar data were obtained following infection at MOI = 5, albeit, where evident, the CPE induction was faster compared to the CPE induction following infection at MOI = 0.1 (data not shown).

### Differential infectivity between RSV A2 and the clinical isolates

**Figure 3:**
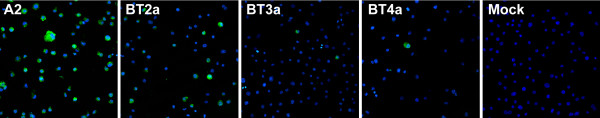
**Differential infectivity between RSV A2, BT2a, BT3a and BT4a**. PBECs were infected with RSV A2, BT2a, BT3a and BT4a (MOI of 0.1) during 2 h and washed with PBS. Seventy two hpi, cells were washed with PBS, fixed with 4% paraformaldehyde and stained for RSV F protein (green) and nuclei (blue). Cultures were observed under a confocal microscope. RSV A2 infected considerably more PBECs than the 3 clinical isolates. Original magnification, x10.

To determine whether the differential CPE between RSV A2 and the clinical isolates was due to their respective capacities to infect PBECs, infected monolayers (MOI = 0.1) were stained for RSV F protein expression. Representative *en-face *micrographs are presented in Figure 3. Consistent with the CPE data, most cells were positive for RSV F protein by 72 hpi with RSV A2, indicating extensive infection and efficient virus propagation. In contrast, but also consistent with the CPE data, few cells were infected with the RSV clinical isolates, with BT2a and BT4a the most and least infectious, respectively.

### RSV A2 and the clinical isolates have differential growth kinetics in PBECs

**Figure 4:**
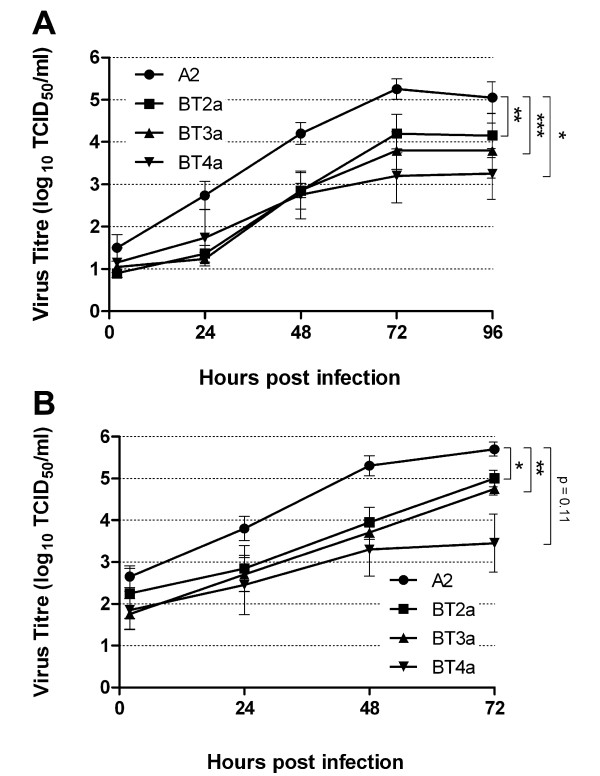
**RSV A2, BT2a, BT3a and BT4a one step and multistep growth curves**. PBEC monolayers from 5 individual donors were infected with RSV A2, BT2a, BT3a and BT4a at MOIs of 0.1 (A) or 5 (B). Cells were scraped into the medium and harvested every 24 h post infection. Virus growth kinetics were determined by titrating virus in each sample. The data are presented as mean ± S.E. log_10 _TCID_50_/ml. Areas under the curve were calculated and compared using a student's paired t-test. *P < 0.05. **P < 0.01. ***P < 0.001.

To address the relative growth kinetics of each virus, PBEC monolayers (n = 5) were inoculated with each RSV strain at MOIs of 0.1 and 5 to establish multistep and one-step growth curves, respectively. Samples were harvested at several time points post-infection, beginning at 2 h. As expected, RSV A2 grew faster and to higher titers following infection at both MOIs than the clinical isolates (Figure 4), while area-under-the-curves were also significantly higher for A2 compared to the clinical isolates (p < 0.05), with the exception of BT4a at MOI 5. Average RSV A2 titers peaked at 5.25 and 5.70 log_10 _TCID_50_/ml for MOIs of 0.1 and 5, respectively. In comparison, peak average titers for RSV BT2a, BT3a and BT4a after infection were 4.20, 3.80 and 3.25 log_10 _TCID_50_/ml at MOI = 0.1, respectively, and 5.00, 4.75 and 3.45 log_10 _TCID_50_/ml at MOI = 5, respectively. These relatively high titers, especially for BT2a and BT3a at MOI = 5, were unexpected and inconsistent with the observed CPE and infectivity data for each of these viruses.

### Multiple passaging of RSV BT2a does not affect growth kinetics

**Figure 5:**
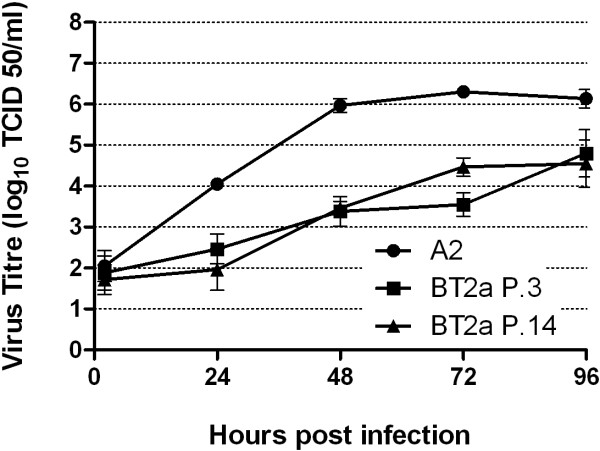
**RSV A2, BT2a P.3 and BT2a P.14 multistep growth curves**. PBEC monolayers from 3 individual donors were infected with RSV A2, BT2a passage 3 and BT2a passage 14 at MOI 0.1 to determine if multiple passages of a clinical isolate in HEp-2 cells influence growth kinetics of the clinical isolates in PBECs. Cells were scraped into the medium and harvested every 24 h post infection. Virus growth kinetics were determined by titrating virus in each sample. The data are presented as mean ± S.E. log_10 _TCID_50_/ml.

As a prototypic strain, RSV A2 is likely to have been passaged many times *in vitro *in continuous cell monolayers and adapted to these conditions. To address whether multiple passaging *in vitro *might explain the differential growth kinetics between RSV A2 and the clinical isolates, RSV BT2a was blindly passaged 14 times in HEp-2 cells. The resultant virus stock (BT2a P.14) was infected onto PBEC cultures (n = 3), in parallel with the passage 3 stock (BT2a P.3) and RSV A2, each at an MOI of 0.1. Samples were harvested at 2, 24, 48, 72 and 96 hpi and virus titers determined (Figure 5). As expected, area-under-the-curves were significantly higher for A2 compared to the BT2a P.3 (p = 0.0122). However, there were no significant differences between the growth curves of BT2a P.3 and P.14 stocks (p = 0.2768). Therefore, serial passaging of the RSV clinical isolate, at least to passage 14, is unlikely to explain the differential growth kinetics and, by extension, the cytopathogenesis of the prototypic RSV A2 strain and the clinical isolates.

### Differential chemokine/cytokine secretion following RSV A2 and clinical isolate infection

**Figure 6:**
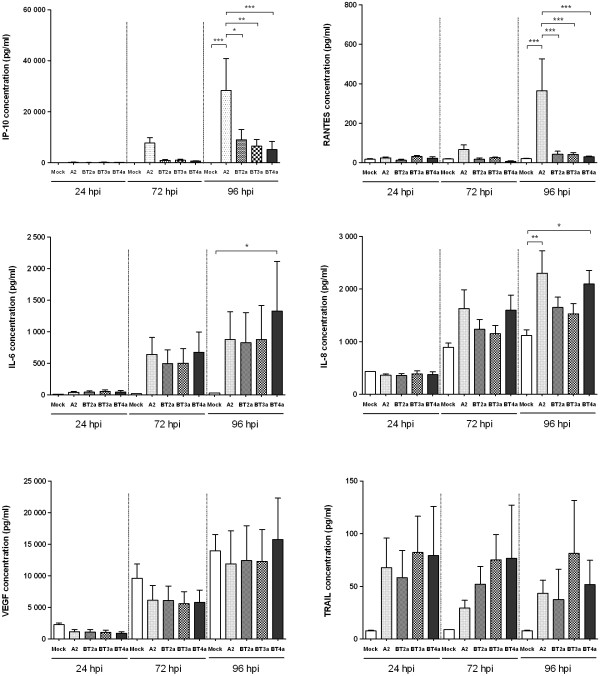
**Chemokines secretion induced following RSV infection**. PBECs were infected with RSV A2, BT2a, BT3a or BT4a at an MOI of 0.1 during 2 h and washed with PBS. Supernatant from infected- and mock-infected cultures were harvested at 24, 72 and 96 h post infection and tested for RANTES (CCL5), IP-10 (CXCL10), IL-6, IL-8 (CXCL8), TNF-related apoptosis-inducing ligand (TRAIL) and vascular endothelial growth factor (VEGF). All values are means of 6 individual donors ± SE. Data were analyzed using One-Way repeated measures ANOVA with a Bonferroni post-test. *P < 0.05. **P < 0.01. ***P < 0.001.

Chemokines/cytokines are important mediators of innate and adaptive immune responses to viral infections. To determine the relative consequence of RSV A2 or clinical isolate infection on their expression, supernatants from RSV-infected (MOI = 0.1) (n = 6) and control cultures (n = 2) were screened for a number of analytes at 24, 72 and 96 hpi. The analytes were chosen because they were either previously associated with RSV disease or had biological properties consistent with RSV pathogenesis [13-17]. Interestingly, there was a trend towards increased concentrations of RANTES, IP-10, IL-6, and IL-8 over time post-infection, irrespective of the virus strain used, compared to mock-infected controls (Figure 6). At 24 hpi with any RSV strain, there was no evidence of upregulation of any analyte. By 72 hpi, mean IP-10, IL-6 and IL-8 concentrations were increased, irrespective of the RSV strains used, although these increases were not statistically significant. By 96 hpi, however, IP-10, in particular, and RANTES were highly upregulated in supernatants from RSV A2-infected cultures compared to both mock- and RSV clinical isolate-infected cultures. While mean IP-10 and RANTES concentrations at 96 hpi in supernatants from cultures infected with the clinical isolates were increased relative to mock-infected cultures, this did not reach significance. Interestingly, IL-8 was significantly upregulated at 96 hpi with RSV A2 and BT4a, while IL-6 was significantly increased only following infection with the latter virus. Mean TRAIL concentrations were higher in RSV- compared to mock-infected cultures at all times, irrespective of the virus strain used. However, statistical significance was not attained due to considerable inter-donor variability. In contrast to the other analytes, mean VEGF concentrations progressively increased to similar levels in supernatants from both infected and mock-infected cultures.

## Discussion

Our work sought to address whether the prototypic RSV strain A2 was representative of recent RSV clinical isolates in terms of cytopathogenesis, infectivity, virus growth kinetics, and pro-inflammatory immune responses. As human airway epithelial cells are the primary targets of RSV infection *in vivo*, we addressed our hypothesis using PBEC monolayers. Our data provided convincing evidence that the prototypic A2 strain demonstrated more cytopathogenicity than the clinical isolates in relation to each parameter tested and, consequently, is poorly representative of them under the experimental conditions outlined. The mechanisms responsible for this differential cytopathogenicity remain to be elucidated and are the subject of ongoing research.

As a prototypic strain, RSV A2 has been extensively passaged *in vitro *in continuous cell lines, such as HEp-2. In contrast, the clinical isolates have not. RSV A2 would therefore be expected to be better adapted to replication in HEp-2 cells than the clinical isolates. Indeed, virus stock titers generated in HEp-2 cells were invariably higher for RSV A2 than for the clinical isolates (data not shown). In contrast, because of their low passage *in vitro*, we expected the clinical isolates to be better adapted than RSV A2 to the PBEC cultures. Consequently, the fact that RSV A2 infected these cells more efficiently and caused considerably more CPE than the recent clinical isolates was unexpected.

The different growth curve slopes between RSV A2 and the clinical isolates, particularly at early time points, are consistent with differential receptor usage between these viruses. Numerous studies have identified heparan sulfate (HS) as essential for RSV entry into continuous cell lines [18-24]. Indeed, HS is found on several different mammalian cell lines and on undifferentiated/monolayer PBECs [25,26]. Efficient infectivity of RSV A2 in our PBEC model is consistent with these reports. In contrast, HS appears to be only present on basal cells in bronchial tissues or in well-differentiated (WD) PBECs, but is not found on apical cells [26,27]. As RSV infection is restricted to apical cells *in vivo *and in WD-PBECs [28-32], HS is unlikely to function as an RSV receptor *in vivo*. By extension, use of HS as a receptor for RSV is likely to be an *in vitro *artifact, to which the clinical isolates have not, or only poorly, adapted in limited passages on HEp-2 cells. Indeed, a relatively low binding affinity to HS might explain the limited infectivity of the clinical isolates in our PBEC model. Experiments are ongoing to address receptor usage, including heparinase treatment of PBEC cultures prior to infection.

It was possible that multiple passaging of RSV A2 in monolayer cells resulted in cell adaptation, thereby explaining the differential infectivity between A2 and the clinical isolates. However, our data did not support this hypothesis, as multiple passaging of RSV BT2a on HEp-2 cells did not substantially alter its phenotype in terms of growth kinetics. It is important to point out, however, that RSV BT2a was passaged blindly. Thus, there was no intentional selection for any phenotypic traits, such as plaque size, that might be expected to alter the virus' characteristics. Alternatively, passaging RSV BT2a 14 times might be insufficient to induce or detect altered phenotypes.

The mean concentrations of IP-10 induced following RSV A2 infection of PBECs were remarkably similar to those reported in intubated children with RSV bronchiolitis [16]. Mean IP-10 induction following infection with the clinical isolates was considerably lower than with A2 but was elevated, although not significantly, in most individuals compared to mock-infected controls. These data indicate that bronchial epithelial cells are a source of IP-10 following RSV infection and suggest that it may be implicated in RSV pathogenesis. Like IP-10, the levels of RANTES secretion following PBEC infection were clearly RSV strain-dependent. The induction of high RANTES levels following RSV A2 infection of PBECs is consistent with previous studies *in vitro *in monolayers [13,14,33]. Elevated RANTES levels were also reported in infants hospitalized with RSV infections [13,16,34]. However, the limited RANTES secretion following PBEC infection with the RSV clinical isolates contrasts with these *in vitro *and *in vivo *data. Our data suggest that the efficiency with which the RSV strains infect and replicate in PBECs dictates the level of secretion of RANTES. Alternatively, if the clinical isolates are considered more representative of RSV infection in humans than RSV A2, our data may be reconciled by the possibility that bronchial epithelial cells are not the principle source of RANTES in RSV-infected individuals.

IL-8 is a potent neutrophil chemoattractant that was previously shown to be secreted following RSV infection *in vitro *[13-15]. In our model, there was a general trend towards increased IL-8 secretion following PBEC infection, irrespective of the virus strain used. However, high IL-8 secretions from mock infected PBECs rendered these increases non-significant in most cases. The high IL-8 expression in mock-infected cultures is consistent with studies in monolayers derived from nasal explants [13] or primary human tracheobronchial epithelial cells [35]. The reasons for elevated IL-8 expression in these primary cultures are not clear. Interestingly, Chang *et al *demonstrated a dose effect of all-trans retinoic acid (ATRA) on IL-8 expression [35]. However, relative to cultures grown in the absence of ATRA, there was no increased IL-8 stimulation evident at doses similar to those used in our PBEC growth medium.

Like IL-8, there were strong trends towards increased IL-6 and TRAIL secretion in RSV-infected PBECs. However, considerable variability among individuals prevented these responses reaching significance. Similarly, there was little or no evidence of RSV strain-dependence on the secretion of these analytes. These data contrast with previous reports in which RSV infection of monolayers resulted in increased IL-6 expression [13,14,33], while work by Bem *et al *suggested that TRAIL might contribute to lung epithelial injury in children with severe RSV infection [17]. Like TRAIL, VEGF is a cytokine associated with RSV or rhinovirus infection in airway bronchial cells grown in monolayer [36] and has been detected in nasopharyngeal aspirates from children hospitalized with severe RSV infection [37]. However, unlike TRAIL and IL-6, in our model, VEGF was secreted at similar levels in both infected and uninfected PBEC cultures. Thus, conclusions regarding a role for IL-6, TRAIL and VEGF in RSV pathogenesis are not possible from the current study.

In conclusion, our data highlight the dramatic cytopathic differences between 3 recent clinical isolates and a prototypic RSV strain in a PBEC infection model. They thereby emphasize the fact that RSV A2 is not necessarily representative of these isolates. Consequently, our findings suggest that the choice of RSV strain may have important implications for future studies on RSV pathogenesis and our understanding of the molecular mechanisms thereof.

## Competing interests

The authors declare that they have no competing interests.

## Authors' contributions

RV generated most of the data for the manuscript and co-wrote the paper. DOD developed the PBEC monolayer model of RSV infection, recruited patients and collected clinical samples, isolated and performed the initial characterization of the clinical RSV strains. ST recruited patients and collected clinical samples. OT was responsible for the molecular characterization of some of the RSV clinical isolates. GS and LHG co-designed and conceived the study and helped with data analyses. JPM supervised the clinical and bronchial sampling aspects of the study and the study feasibility. PVC input to study design and was responsible for the screening of all patients for viral infections. MDS co-designed and conceived the study, obtained research ethics and institutional governance permission, designed and co-supervised the clinical aspects of the study, and helped with data analyses. UFP co-designed, conceived and coordinated the study, and co-wrote the paper. All authors read and approved the final manuscript.
